# Age-specific urinary metabolite signatures and functions in patients with major depressive disorder

**DOI:** 10.18632/aging.102133

**Published:** 2019-09-06

**Authors:** Jian-Jun Chen, Jing Xie, Wen-Wen Li, Shun-Jie Bai, Wei Wang, Peng Zheng, Peng Xie

**Affiliations:** 1 Institute of Life Sciences, Chongqing Medical University, Chongqing 400016, China; 2 Department of Endocrinology and Nephrology, Chongqing University Central Hospital, Chongqing Emergency Medical Center, Chongqing 400014, China; 3 Department of Pathology, Faculty of Basic Medicine, Chongqing Medical University, Chongqing 400016, China; 4 Department of Laboratory, The First Affiliated Hospital of Chongqing Medical University, Chongqing 400016, China; 5 NHC Key Laboratory of Diagnosis and Treatment on Brain Functional Diseases, Chongqing Medical University, Chongqing 400016, China; 6 Department of Neurology, the First Affiliated Hospital of Chongqing Medical University, Chongqing 400016, China; 7 Department of Neurology, Yongchuan Hospital of Chongqing Medical University, Chongqing 400016, China

**Keywords:** major depressive disorder, metabolite, metabolomics, biomarker

## Abstract

Major depressive disorder (MDD) patients in different age ranges might have different urinary metabolic phenotypes, because age could significantly affect the physiological and psychological status of person. Therefore, it was very important to take age into consideration when studying MDD. Here, a dual platform metabolomic approach was performed to profile urine samples from young and middle-aged MDD patients. In total, 18 and 15 differential metabolites that separately discriminated young and middle-aged MDD patients, respectively, from their respective HC were identified. Only ten metabolites were significantly disturbed in both young and middle-aged MDD patients. Meanwhile, two different biomarker panels for diagnosing young and middle-aged MDD patients, respectively, were identified. Additionally, the TCA cycle was significantly affected in both young and middle-aged MDD patients, but the Glyoxylate and dicarboxylate metabolism and phenylalanine metabolism were only significantly affected in young and middle-aged MDD patients, respectively. Our results would be helpful for developing age-specific diagnostic method for MDD and further investigating the pathogenesis of this disease.

## INTRODUCTION

Major depressive disorder (MDD) is a seriously debilitating mental illness that can cause huge economic burden to families and society. The quality of life of many people is seriously affected by this disease. MDD affects up to 15% the total population worldwide and is a vital cause of disability [1]. MDD patients with suicidal ideation have a higher rate of suicide. Previous studies reported that about 50% of MDD patients experienced suicidal ideation in the week prior to attempting suicide [2, 3]. However, no objective method is available to diagnose MDD nowadays. Its diagnosis mainly relies on the clinical features and symptomatic clusters. But this method results in a high rate of misdiagnosis and under-diagnosis, due to the high heterogeneity of depressive symptoms [4]. Thus, it is urgently needed to identify objective biomarkers for MDD.

Metabolomics can quantitatively measure the endogenous metabolites in any given samples, such as urine and plasma. It has been widely used to choose disease-specific metabolites as potential biomarkers [5, 6]. Currently, there are three main kinds of analytical technologies using for non-targeted metabolomic profiling: gas and liquid chromatography mass spectroscopy (GC-MS, LC-MS), and nuclear magnetic resonance (NMR). Many researchers have successfully used these analytical technologies to identify some biomarkers for various neuropsychiatric disorders, such as schizophrenia and autism [7, 8]. However, each analytical technology has its own advantages and disadvantages. Thus, the use of multiple analytical technologies could allow researchers to identify some unknown urine metabolites [9, 10]. In our previous study, the combined application of GC-MS and NMR yielded a more comprehensive metabolite panel for diagnosing bipolar disorder [11].

Our group has also used metabolomics to identify some possible biomarkers for MDD [12, 13]. However, our previous studies did not take age into consideration. MDD patients in different age ranges might have different urinary metabolic phenotypes, because age could significantly affect the physiological and psychological status of person [14]. As a special factor, age has the characteristics of inevitable and internal steady growth in the lifetime of humans. In the three different key phases of life cycle (young, middle-age and old), people present the different biological characteristics [15]. Some studies have found that age could affect the composition of gut microbiome [16, 17]. Chia et al. also reported that age could affect the levels of metabolites [15]. Meanwhile, our previous work found that the disturbed gut micorbiota had a close relationship with depression [18, 19]. Therefore, investigating the different characteristics of urinary metabolites in particular age phases could improve our understanding of the underlying pathogenesis of MDD. In this study, the combined application of NMR and GC-MS was used to explore the urinary metabolite signatures and functions in young and middle-aged patients with MDD.

## RESULTS

### Building discrimination model

**Figure 1:**
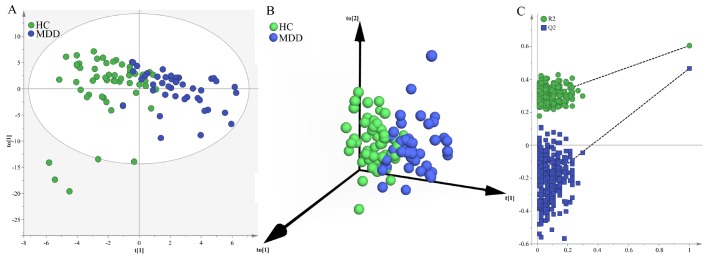
**Metabolomic analysis of urine samples from the young populations.** (**A**) OPLS-DA model showed an obvious separation between young HCs (green circle) and young MDD patients (blue circle); (**B**) 3D view also showed an obvious separation between young HCs (green sphere) and young MDD patients (blue sphere); (**C**) the permutation test suggested the validity of the model, as the Q2 and R2 values yielded by the permutation test (left bottom) were lower than their original values (upper right).

Firstly, we used the samples in the young group to build OPLS-DA model. The built model showed that young MDD patients and young HCs could be significantly separated with little overlap (R^2^Y=61%, Q^2^Y=47%; Figure 1A and 1B). The 50 of the 56 young HCs and 40 of the 44 young MDD patients were correctly diagnosed. The positive values of R^2^Y and Q^2^Y indicated that there was a robust metabolic difference in young MDD patients compared to young HCs. Moreover, the permutation test suggested that the built model was valid and not over-fitted (Figure 1C).

**Figure 2:**
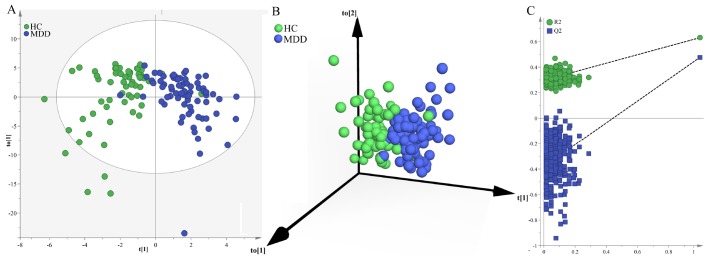
**Metabolomic analysis of urine samples from the middle-aged populations.** (**A**) OPLS-DA model showed an obvious separation between middle-aged HCs (green circle) and middle-aged MDD patients (blue circle); (**B**) 3D view also showed an obvious separation between middle-aged HCs (green sphere) and middle-aged MDD patients (blue sphere); (**C**) the permutation test suggested the validity of the model, as the Q2 and R2 values yielded by the permutation test (left bottom) were lower than their original values (upper right).

Secondly, we used the samples in the middle-aged group to build OPLS-DA model. The built model showed that middle-aged MDD patients and middle-aged HCs could be significantly separated with little overlap (R^2^Y=63%, Q^2^Y=48%; Figure 2A and 2B). The 54 of the 61 middle-aged HCs and 68 of the 74 middle-aged MDD patients were correctly diagnosed. The positive values of R^2^Y and Q^2^Y indicated that there was a robust metabolic difference in middle-aged MDD patients compared to middle-aged HCs. Moreover, the permutation test suggested that the built model was valid and not over-fitted (Figure 2C).

### Identifying differential metabolites

**Figure 3:**
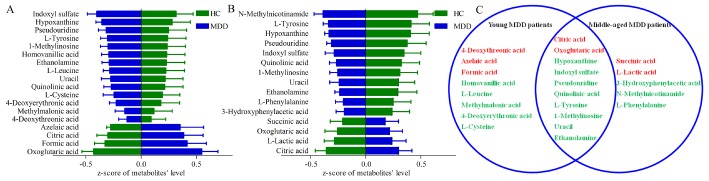
**Differential urinary metabolites between the different groups.** (**A**) 18 differential metabolites between young HCs and young MDD patients; (**B**) 15 differential metabolites between middle-aged HCs and middle-aged MDD patients; (**C**) eight and five metabolites were only altered in young and middle-aged MDD patients, respectively, and 10 metabolites were altered in both young and middle-aged MDD patients.

By analysis of OPLS-DA loadings plot and nonparametric Mann-Whitney *U* test, we identified 18 differential metabolites (VIP>1.0 and adjusted *p*-value<0.05) (14 down-regulated and 4 up-regulated) responsible for the discrimination between young MDD patients and young HCs (Figure 3A), and 15 differential metabolites (11 down-regulated and 4 up-regulated) responsible for the discrimination between middle-aged MDD patients and middle-aged HCs (Figure 3B). As shown in Figure 3C, ten metabolites were significantly changed (eight down-regulated and two up-regulated) in both young and middle-aged MDD patients. These differential metabolites were mainly related with amino acid metabolism and lipid metabolites.

### Significantly affected pathways identification

**Figure 4:**
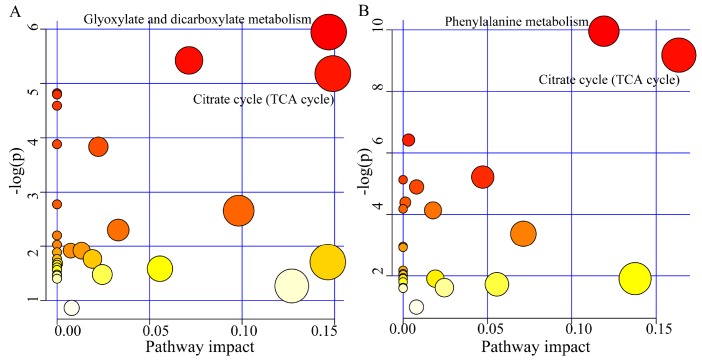
**Significantly affected pathways founded using online software MetaboAnalyst 3.0 (*p*-value<0.05, impact>0.1).** (**A**) Glyoxylate and dicarboxylate metabolism and Citrate cycle (TCA cycle) was significantly affected in young MDD patients; (**B**) Phenylalanine metabolism and Citrate cycle (TCA cycle) was significantly affected in middle-aged MDD patients.

The pathway analysis was conducted using the online software MetaboAnalyst 3.0. The results showed that there were two significantly affected pathways in young MDD patients (p<0.05, impact>0.1): Glyoxylate and dicarboxylate metabolism (p=0.002, impact=0.146) (Oxoglutaric acid, Formic acid and Citric acid) and Citrate cycle (TCA cycle) (p=0.005, impact=0.149) (Oxoglutaric acid and Citric acid) (Figure 4A); there were also two significantly affected pathways in middle-aged MDD patients: Phenylalanine metabolism (p=0.00004, impact=0.119) (L-Phenylalanine, Succinic acid, L-Tyrosine, and 3-Hydroxyphenylacetic acid) and Citrate cycle (TCA cycle) (p=0.001, impact=0.163) (Succinic acid, Citric acid and Oxoglutaric acid) (Figure 4B).

### Simplified biomarker panel identification and assessment

**Figure 5:**
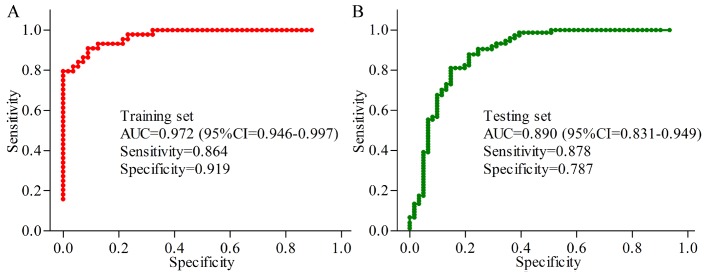
**Quantification of the diagnostic performance of the two identified biomarker panels.** (**A**) an area under the curve (AUC) of 0.972 in young populations; (**B**) an AUC of 0.890 in middle-aged populations.

In the young group, the results of logistic-regression analysis showed that the most significant deviations between MDD patients and HCs could be described by six urinary metabolites: Oxoglutaric acid, 4-Deoxythreonic acid, Azelaic acid, Hypoxanthine, Indoxyl sulfate and L-Tyrosine. The ROC curve analysis found that the simplified biomarker panel consisting of these metabolites could effectively diagnose 44 MDD patients from 56 HCs with AUC of 0.972 (Figure 5A). In the middle-aged group, the simplified biomarker panel was consisting of Succinic acid, L-Tyrosine, N-Methylnicotinamide, Hypoxanthine, Indoxyl sulfate and L-Phenylalanine. The ROC curve analysis found that this panel could effectively diagnose74 MDD patients from 61 HCs with AUC of 0.890 (Figure 5B).

### Excellent diagnostic performance of age-specific biomarkers

Meanwhile, the other biomarkers panel of MDD identified by GC-MS [12] was used to predict the young and middle-aged populations. We found that these biomarkers panel only yielded an average accuracy of 76.7% in the young population and 73.8% in the middle-aged population. Next, the other biomarkers panel of MDD identified by NMR [13] was used to predict the young and middle-aged populations. We found that these biomarkers panel only yielded an average accuracy of 79.0% in the young population and 68.9% in the middle-aged population. These results indicated that the average accuracy obtained using these biomarkers are lower than those obtained using the age-specific biomarkers (young: 89.0%; men: 83.7%). In addition, the average accuracy of 89.0% in the young population yielded by the young panel was higher than the average accuracy of 78.5% obtained using the middle-aged panel to predict the young population; the average accuracy of 83.7% in the middle-aged population yielded by the middle-aged panel was higher than the average accuracy of 75.4% obtained using the young panel to predict the middle-aged population.

### Sex-specific urinary differences in different age populations

**Figure 6:**
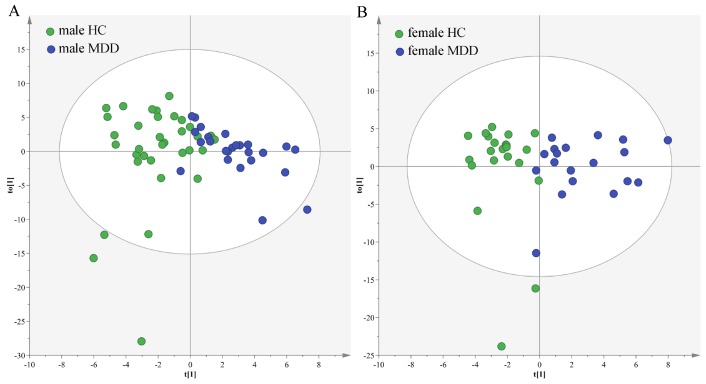
**Sex-specific urinary differences in young populations.** (**A**) OPLS-DA model showed an obvious separation between male HCs (green circle) and male MDD patients (blue circle); (**B**) OPLS-DA model showed an obvious separation between female HCs (green circle) and female MDD patients (blue circle).

In the young populations, we used the data from the male HCs and male MDD patients to build OPLS-DA model. The model showed that the two groups could be obviously separated with little overlap (Figure 6A). There were 22 differential metabolites (VIP>1.0 and adjusted *p*-value<0.05): Isobutyric acid, Hypoxanthine, L-Alanine, α-Hydroxyisobutyric acid, Succinic acid, Acetamide, Pyruvic acid, 1-Methylinosine, Indoxyl sulfate, Acetic acid, L-Tyrosine, Pseudouridine, Quinolinic acid, Trimethylamine, Formic acid, 2-ketoglutaric acid, Methylmalonic acid, α-Hydroxybutyric acid, Oxoglutaric acid, Ethanolamine, L-Phenylalanine and 4-Hydroxybenzoic acid. The former 15 differential metabolites were also identified in our previous study, which focused on sex-specific urinary differences for MDD [20]. Meanwhile, we used the data from the female HCs and female MDD patients to build OPLS-DA model. The model showed that the two groups could be obviously separated with little overlap (Figure 6B). There were 14 differential metabolites (VIP>1.0 and adjusted *p*-value<0.05): L-Lactic acid, L-Alanine, Methylmalonic acid, Azelaic acid, Indoxyl sulfate, Oxoglutaric acid, α-Hydroxyisobutyric acid, Succinic acid, Acetic acid, Citric acid, Pyruvic acid, α-Hydroxybutyric acid, Acetamide and Glycine. The former five differential metabolites were also identified in our previous study [20].

**Figure 7:**
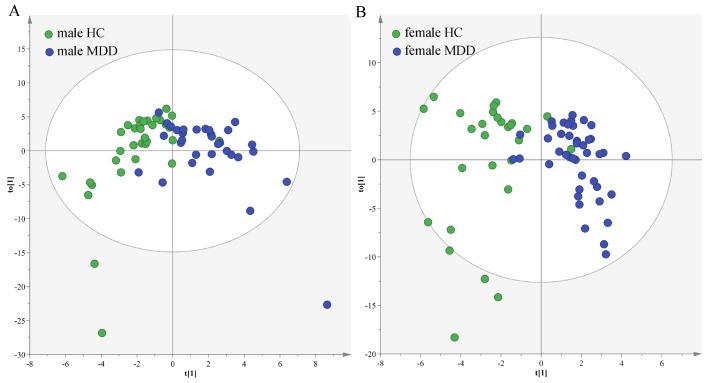
**Sex-specific urinary differences in middle-aged populations.** (**A**) OPLS-DA model showed an obvious separation between male HCs (green circle) and male MDD patients (blue circle); (**B**) OPLS-DA model showed an obvious separation between female HCs (green circle) and female MDD patients (blue circle).

In the middle-aged population, we used the data from the male HCs and male MDD patients to build OPLS-DA model. The model showed that the two groups could be obviously separated with little overlap (Figure 7A). There were 14 differential metabolites (VIP>1.0 and adjusted *p*-value<0.05): Hypoxanthine, N-Methylnicotinamide, L-Tyrosine, Indoxyl sulfate, 1-Methylinosine, Pseudouridine, Dimethylglycine, m-Hydroxyphenylacetic acid, Trimethylamine N-oxide, 4-Deoxythreonic acid, Glyceroylphosphocholine, Malonic acid, 3-Hydroxyphenylacetic acid and Dimethylamine. The former six differential metabolites were also identified in our previous study, which focused on sex-specific urinary differences for MDD [20]. Meanwhile, we used the data from the female HCs and female MDD patients to build OPLS-DA model. The model showed that the two groups could be obviously separated with little overlap (Figure 7B). There were nine differential metabolites (VIP>1.0 and adjusted *p*-value<0.05): Hypoxanthine, N-Methylnicotinamide, Azelaic acid, Methylmalonic acid, Citric acid, Oxoglutaric acid, Glycine, 4-Deoxythreonic acid and Formic acid. The former four differential metabolites were also identified in our previous study [20].

## DISCUSSION

This is the first study to explore the urinary metabolite signatures and functions in young and middle-aged patients with MDD using the multiple metabolomics platforms. We successfully found 18 and 15 differential metabolites in urine samples of young and middle-aged MDD patients, respectively. Furthermore, we identified two simplified biomarker panels for diagnosing young MDD patients (Oxoglutaric acid, 4-Deoxythreonic acid, Azelaic acid, Hypoxanthine, Indoxyl sulfate and L-Tyrosine) and middle-aged MDD patients (Succinic acid, L-Tyrosine, N-Methylnicotinamide, Hypoxanthine, Indoxyl sulfate and L-Phenylalanine). The AUC of young and middle-aged panels were 0.972 and 0.890, respectively. These results demonstrated that these two simplified biomarker panels might be the “good” classifier of young and middle-aged MDD patients and their respective HCs, and these potential biomarkers could be helpful for developing the objective diagnostic methods.

Manuela et al. found that age and sex-related physiological conditions could be reflected in the metabolome of healthy humans, and the plasma and urine metabolite profiles could predict the sex and age with high accuracy [21]. Another study reported that the metabolite levels were different between longer- and shorter-lived mammalian species [22]. In this study, we found that the young and middle-aged MDD patients had divergent urinary metabolic phenotypes. In this study and our previous study [20], we found that male and female MDD patients have distinct metabonomic signatures. These studies suggested that the age and sex were associated with different metabolic phenotypes in humans, and therefore should be considered in metabolomics studies.

**Table 1:** Demographic and clinical characteristics of MDD patients and HCs^a^.

	**Young group (18–29 years)**			**Middle-aged group (30–59 years)**		
	**HC**	**MDD**	***p*-value**	**HC**	**MDD**	***p*-value**
**Sample Size**	56	44	–	61	74	–
**Age (years)^c^**	23.95±2.45	23.32±3.17	0.22	41.49±7.67	39.66±6.57	0.14
**Sex (female/male)**	22/34	19/25	0.69	28/33	42/32	0.21
**BMI**	21.30±2.62	22.30±2.83	0.70	21.71±2.88	21.50±2.78	0.66
**HDRS scores**	2.09±1.44	21.40±3.67	<0.00001	2.36±1.52	23.18±4.35	<0.00001

^***a***^Abbreviations: MDD: major depressive disorder; HC: healthy control; BMI: body mass index; HDRS: Hamilton Depression Rating Scale

**Table 2:** Key urinary metabolites responsible for the discrimination of MDD patients and HCs.

**Metabolites**	**VIP**	***p*-value**	**Adjusted *p*-value**	**Fold change**	**Platform**
***Young***					
Homovanillic acid	1.15	0.014	0.045	1.76	GC-MS
L-Leucine	1.17	0.014	0.046	1.65	GC-MS
4-Deoxythreonic acid	1.06	8.49E-06	0.00018	1.64	GC-MS
4-Deoxyerythronic acid	1.09	0.000075	0.0011	1.61	GC-MS
Methylmalonic acid	1.04	0.0039	0.015	1.54	GC-MS
L-Cysteine	1.11	0.0024	0.011	1.44	GC-MS
Formic acid	1.23	0.005	0.019	0.68	NMR
Azelaic acid	1.06	0.000057	0.001	0.17	GC-MS
***Young/Middle-aged***					
1-Methylinosine	1.22/1.31	0.00034/0.00025	0.0027/0.0013	2.15/1.99	GC-MS
Citric acid	1.23/1.23	0.00027/0.002	0.002/0.0072	0.68/0.72	NMR
Ethanolamine	1.29/1.24	0.011/0.00098	0.038/0.0043	2.02/1.89	GC-MS
Hypoxanthine	1.22/1.64	2.79E-10/1.09E-08	2.45E-07/4.83E-06	3.79/3.59	GC
Indoxyl sulfate	1.29/1.34	0.00011/0.000039	0.0012/0.00028	2.06/1.76	GC
L-Tyrosine	1.17/1.56	2.96E-06/0.0015	8.6E-05/0.006	2.39/2.95	GC
Oxoglutaric acid	1.74/1.06	0.008/4.97E-07	0.03/7.28E-06	0.75/0.86	NMR
Pseudouridine	1.21/1.48	0.00013/0.00023	0.0013/0.0012	2.2/2.29	GC
Quinolinic acid	1.15/1.41	0.00054/0.0034	0.0036/0.011	1.94/1.96	GC
Uracil	1.24/1.39	0.0026/0.0023	0.011/0.0077	1.9/1.71	GC
***Middle-aged***					
N-Methylnicotinamide	1.83	0.001	0.0042	2.08	NMR
L-Phenylalanine	1.32	0.01	0.028	1.52	GC-MS
L-Lactic acid	1.21	1.12E-06	0.000014	0.8	NMR
Succinic acid	1.05	0.000015	0.00015	0.86	NMR
3-Hydroxyphenylacetic acid	1.03	0.0046	0.013	1.94	GC-MS

^a^*p*-value was obtained from the nonparametric Mann-Whitney *U* test.

^b^adjusted *p*-value was obtained using Benjamini and Hochberg False Discovery Rate.

^c^values less than 1.0 indicated significantly lower levels in MDD patients; values higher than 1.0 indicated significantly higher levels in MDD patients.

Metabolomics could unbiasedly measure the small molecules in samples such as tissues, cells and biological fluid. Many factors can influence the levels of metabolites in biosamples, including drugs, dietary habit, and disease status. Age is definitely an important factor [15]. Previous study reported that the serum nitrite and nitrate levels in different age groups were different [23]. Hedner et al. found that the homovanillic acid, 3-methoxy-4-hydroxyphenyl glycol and 5-hydroxyindoleacetic acid in cerebrospinal (CSF) fluid were significantly higher in early infancy than in adolescence [24]. Another study reported that the levels of some urinary metabolites changed with age [25]. Here, we found that the levels of almost half of the differential metabolites were only significantly changed in young MDD patients, and 1/3 of the differential metabolites were only significantly changed in middle-aged MDD patients. Meanwhile, the young and middle-aged biomarker panel shared only half of potential biomarkers. In addition, we found that the Glyoxylate and dicarboxylate metabolism and phenylalanine metabolism were only significantly affected in young and middle-aged MDD patients, respectively. These findings showed that understanding the different characteristics of urinary metabolites in different age phases of MDD patients might be facilitated to study the pathogenesis of MDD.

As a vital metabolic network in oxidative organisms, TCA cycle has two important roles in metabolism [26]. First, it is responsible for the total oxidation of acetyl-CoA under aerobic conditions; the acetyl-CoA is mainly derived from the pyruvate that is produced by glycolysis. Second, the biosynthesis of several amino acids requires its intermediates. In the chronic mild stress rat model of depression, we found that the TCA cycle was disturbed in the cerebellum tissue [27]. In our previous metabolomics studies, some TCA cycle-associated metabolites were significantly perturbed in MDD patients [12, 13]. In this study, we found that the TCA cycle was significantly affected in both young and middle-aged MDD patients. These consistent set of findings demonstrated that MDD was associated with disturbances in TCA cycle.

The TCA cycle is associated with Glyoxylate and dicarboxylate metabolism via a connection with oxaloacetate. In this study, the Glyoxylate and dicarboxylate metabolism was found to be significantly affected only in young MDD patients. Both metabolism pathways are closely related with energy metabolism [28, 29], which points to a disorder of energy regulation in young MDD patients. Phenylalanine is an essential precursor of many aromatic compounds and also an important component of body proteins. Together with tyrosine, phenylalanine is involved in the synthesis of important neurotransmitters. Previous study reported that the depressive behaviors of chronic unpredictable mild stress rats are associated with the levels of metabolites in phenylalanine metabolism [30]. Our previous work also found that some phenylalanine metabolism-associated metabolites were significantly perturbed in adult depressed rats [31]. Here, we found the disturbed phenylalanine metabolism only in middle-aged MDD patients. Limited by the relatively small samples, these findings were needed future studies to validate.

Limitations of this work included: i) the number of samples here was relatively small; but the high AUC values suggested the good representativeness of these differential metabolites; ii) all samples came from the same place, which possibly limited the general applicability of our results [32–34]; iii) other biosampels, such as CSF and brain tissue, should be explored to ensure our differential urinary metabolites being relevant to the pathogenesis of MDD; iv) only two key phases of life cycle (young and middle-age) were investigated here; future studies were needed to study the change trend of our differential urinary metabolites in old MDD patients.

In conclusion, we successfully identified different urinary metabolic phenotypes in young and middle-aged MDD patients. Only ten metabolites were significantly disturbed in both young and middle-aged MDD patients. Meanwhile, two effective biomarker panels for diagnosing young and middle-aged MDD patients were identified. Additionally, we found that the TCA cycle was significantly affected in both young and middle-aged MDD patients, but the Glyoxylate and dicarboxylate metabolism and phenylalanine metabolism were only significantly affected in young and middle-aged MDD patients, respectively. Our results would be helpful for developing age-specific diagnostic method for MDD and investigating the pathogenesis of this disease.

## MATERIALS AND METHODS

### Subject recruitment

Ethical Committee of Chongqing Medical University reviewed and approved this study. In total, there were 44 MDD patients (age 18-29 years) and 56 demographics-matched healthy controls (HCs) (age 20-29 years) in the young group; there were 74 MDD patients (age 30-58 years) and demographics-matched 61 HCs (age 30-59 years) in the middle-aged group. These MDD patients were recruited from the psychiatric center of our hospital. The HCs were recruited from the Medical Examination Center of our hospital. Two experienced psychiatrists systematically used the Hamilton Depression Rating Scale (HDRS) to review each patient. Patients with HDRS score >=17 were included in this study. MDD patients with other mental disorders or illicit drug use were excluded, and HCs with DSM-IV Axis I/II disorders or systemic medical illness were excluded. Meanwhile, the history of neuropsychiatric disorder in first-degree relatives and MDD history was also the exclusion criteria for subjects in this study.

### Sample preparation and metabolites acquisition

After the included subjects provided the written informed consents, morning urine samples were collected using the sterile cup and then transferred into the sterile tube. The urine samples were centrifuged at normal temperature (25°C±1) for 15 min at 1500g. The obtained supernatant was quickly divided into equal aliquots and then stored at –80°C under standard conditions (make a mark, –80°C±10, daily maintenance, set high and low temperature alarm) until later analysis. In this study, the procedure of metabolites acquisition using NMR and GC-MS were exactly performed according to our previous studies [12, 13].

Briefly, the procedure of NMR was: thawed and centrifuged (1500g for 10minutes) the urine samples; added 100μl of phosphate buffer (90% D2O, 1 mM 3-trimethylsilyl-1-[2, 2, 3, 3-²H4] propionate (TSP), and 3 mM sodium azide; pH 7.4) to ensure stabilization of urinary pH (500μl urine); after centrifugation (12000rpm for 10minutes), transferred 500μl supernatant to 5mm NMR tubes. The Bruker Avance 600 spectrometer was used to obtain the proton spectra of the urine samples. The procedure of NMR was: vortexed 15μl urine and 10μl internal standard solutions (L-leucine-13C6, 0.02 mg/ml); added 15μl urease into this mixed solution to degrade the urea (60 minutes at 37°C); extracted the mixture sequentially using 240μl and 80μl ice-cold methanol; after vortexing and then centrifugation (14000rpm for 5minutes at 4°C), transferred 224μl obtained supernatant into glass vial and vacuum-dried at room temperature; derivatized the dried metabolic extract using 30μl methoxyamine (20 mg/ml) (1.5hours at 37°C); added 30μl BSTFA with 1% TCMS into the mixture and then heated (one hour at 70°C); injected 1.0μl obtained trimethylsilyl (TMS) derivatives into GC/MS system (Agilent 7980 GC system).

### Statistical analysis

We used the creatinine to normalize the original spectral data of urinary metabolites to alleviate the effects of the different samples, and scaled the obtained new data to zero-mean and unit-variance to eliminate the effects of different orders of magnitude. Firstly, the obtained metabolites were used to build the orthogonal partial least-squares discriminant analysis (OPLS-DA) model. The OPLS-DA model was used to visualize the discrimination between MDD patients and HCs [35]. The R^2^Y and Q^2^Y resulted from the leave-one-out procedure were used to assess the goodness-of-fit and predictability of the model, respectively [36]. Meanwhile, to check whether or not there was a non-randomness of separation between MDD patients and HCs, the permutation test (399-iteration) was performed [37]. If the Q^2^ and R^2^ values yielded by the permutation test were lower than their original values, then the model was considered valid. These analyses were conducted using SIMCAP+ 14.0 software.

Secondly, by analysis of OPLS-DA loadings plot, the metabolites with variable importance in projection (VIP)>1.0 were viewed as the important metabolites responsible for the discrimination between MDD patients and HCs. Meanwhile, the nonparametric Mann-Whitney *U* test and Benjamini and Hochberg False Discovery Rate were used to check whether or not these identified important metabolites were still significantly different between MDD patients and HCs. The metabolites with VIP>1.0 and adjust *p*-value <0.05 were identified as the differential metabolites between MDD patients and HCs. Then, the online software MetaboAnalyst 3.0 was further used to analyze the pathways affected by the identified differential metabolites.

Finally, the step-wise logistic-regression analysis based on the Akaike’s information criterion (AIC) rule was used to further analyze the identified differential metabolites. The purpose was to obtain a simplified biomarker panel, which would be more convenient and feasible in clinical practice [38]. For a given set of data, the AIC could estimate the quality of each model. It could take the simplicity and the goodness of fit of the model into account at the same time. The preferred model was the model with the minimum AIC value. Thus, it was usually used to perform model selection. Then, the receiver operating characteristic (ROC) curve analysis was used to assess the potential diagnostic efficiency of this panel in discriminating MDD patients from HCs. The area under the curve (AUC) was used as the evaluation index [39]. These analyses were conducted using SPSS 19.0 software.
